# National Black HIV/AIDS Awareness Day — February 7, 2014

**Published:** 2014-02-07

February 7 is National Black HIV/AIDS Awareness Day, an observance intended to raise awareness of human immunodeficiency virus/acquired immunodeficiency syndrome (HIV/AIDS) and encourage action to reduce the disproportionate impact of HIV/AIDS on blacks in the United States. Compared with other races and ethnicities, blacks had the highest HIV incidence in 2010, with an estimated rate of 68.9 per 100,000 population, nearly eight times the estimated rate of 8.7 among whites (1).

By the end of 2010, an estimated 506,800 blacks were living with HIV in the United States, accounting for the highest percentage (44.3%) of persons living with HIV, followed by whites (33.0%), and Hispanics (19.3%) (2).

Information regarding National Black HIV/AIDS Awareness Day is available at http://www.cdc.gov/features/blackhivaidsawareness. Information regarding blacks and HIV/AIDS is available at http://www.cdc.gov/hiv/risk/racialethnic/aa/index.html.
